# Assessment and Treatment of Pain in Hospitalized Children at a Tertiary Children’s Hospital: A Cross-Sectional Mixed Methods Survey

**DOI:** 10.3390/children11070874

**Published:** 2024-07-19

**Authors:** Nadia Roessler De Angulo, Andrea C. Postier, Lisa Purser, Lena Ngo, Karen Sun, Stefan Friedrichsdorf

**Affiliations:** Stad Center for Pediatric Pain, Palliative & Integrative Medicine, Benioff Children’s Hospital, Department of Pediatrics, University of California San Francisco, 1855 Fourth St., 4th Floor, San Francisco, CA 94158, USA; andrea.postier@ucsf.edu (A.C.P.);

**Keywords:** hospital medicine, analgesia, pain, pediatrics, integrative medicine, needles, hospital medicine, multimodal pain treatment

## Abstract

(1) Background: Acute pain in hospitalized children remains under-recognized and under-treated. Our objective is to benchmark pain assessment, documentation, treatment, and patient experience in children admitted to a US children’s hospital. (2) Methods: A cross-sectional, mixed-method survey of pain for children hospitalized ≥24 h. Charts were reviewed for modalities of pain assessment and treatment for all inpatients. If pain was documented, patients/caregivers were surveyed regarding their experience with pain and its management. (3) Results: Chart review: All 107 patients had ≥1 pain score documented. A total of 47 patients had a pain score ≥0, 35 (74.5%) of whom had ≥1 moderate-severe score. Seventy (65.4%) patients received ≥1 intervention for pain, including medications from ≥1 class (e.g., opioids) (*n* = 55, 51.4%) and/or integrative/non-pharmacologic intervention(s) *(n* = 39, 36.4%). There were assessment and documentation gaps. Patient survey: A total of 39 (83.0%) interviews were attempted; 25 (53.2%) were completed. The worst pain was mostly caused by acute illness (*n* = 13, 52%) and painful procedures (*n* = 10, 40%). Suggestions for improvement included increasing the use of integrative modalities and optimizing patient–clinician communication. (4) Conclusions: All patients admitted ≥24 h had ≥1 pain score documented; however, gaps in documentation were common. Multimodal treatment and integrative modalities were underutilized. Procedures were a frequent cause of under-treated pain, prompting an institution-wide quality improvement project.

## 1. Introduction

Many hospitalized infants and children experience pain during hospitalization [1,2,3,4,5,6,7,8,9,10,11], resulting in immediate and long-term consequences. Unrelieved pain puts children at risk for distress, medical complications, increased and harder-to-relieve pain with future procedures or admissions, medical trauma, needle phobia, and development of chronic pain [12,13,14,15,16], which can negatively impact their future interactions with the healthcare system (e.g., vaccine non-adherence) [14,15]. The high stakes of untreated pain in hospitalized children have become increasingly recognized over the past two decades, with major health organizations joining the call for pain recognition and treatment [17,18]. Pain assessment, documentation, and management in hospitalized patients have subsequently become integral to national, state, and local guidelines and policies in the US and are a featured component of hospital accreditation standards [19,20].

Despite the increased recognition of pain underassessment, poor documentation, and under-treatment of pain in hospitalized children, suboptimal pain management remains common. Over the past 30 years, several surveys conducted in pediatric hospitals around the world have affirmed this finding despite mounting evidence on how to recognize, prevent, and treat pain [1,2,3,4,5,6,7,8,9,10,11,21,22]. Interestingly, most of these findings originate from academic tertiary centers with stringent evidence-based pain policies, developmentally appropriate pain assessment tools, and specialized pediatric pain consultation teams. Improvements in pain management have been reported following a QI initiative at a US children’s hospital [23,24].

Acknowledging recent reports of continued suboptimal pain control, the purpose of this chart review and patient survey was to benchmark pain assessment, documentation, management, and patient experience during a 24-h period in hospitalized children at a single tertiary institution to help inform our own quality improvement efforts, and prompt readers to examine practices at their own institution.

## 2. Materials and Methods

### 2.1. Survey Design and Participants

This single-center, cross-sectional, mixed-method survey was conducted to characterize pain assessment, documentation, treatment, and patient experiences for all children admitted to the 183-bed University of California San Francisco (UCSF) Benioff Children’s Hospital (UCSF IRB exemption #21-33485). The survey was administered during two unannounced consecutive weekdays in June 2018 as part of a QI project conducted by the Pediatric Pain, Palliative, and Integrative Medicine (PPPIM) service to identify areas for improvement and provide rapid, tailored feedback to each unit.

### 2.2. Existing Pain Policies

Currently, policies at our institution include the following: a comprehensive pain history and assessment within 24 h of admission (including whether or not the patient has pain in daily life, how they express pain, what makes pain worse, and what alleviates pain); focused pain assessments and documentation of vital signs, before and after any pain treatment, and as needed based on patient condition. Nursing staff are trained to use clinically appropriate scales in each unit: the Neonatal Pain, Agitation, and Sedation Scale (NPASS) [25,26] in the neonatal intensive care unit and the pediatric intensive care unit (ICU); the Neonatal Infant Pain Scale (NIPS) [27] in term newborns to less than 1 year of age; the revised Face, Legs, Activity, Cry, and Consolability Scale (r-FLACC) [28] in pediatric patients ages 2 months to 18 years of age who are unable to self-report pain; the Wong-Baker FACES^®^ Pain Rating Scale (FPS-R) [29] in patients aged ≥3 years to adult and able to recognize faces; the Verbal Descriptor Scale in patients >6 years and able to understand terms mild, moderate, and severe as comparisons; and the Numeric Rating Scale (NRS) [30] in patients aged ≥6 years to adult who understand numeric comparisons. On some units, different scales may be utilized for the same patient depending on their unique needs (e.g., ability to self-report, availability of caregivers for proxy reporting, clinical circumstances, and care needs). Pain re-assessment and documentation are required as follows: within 90 min following administration of oral, rectal, or enteral administered analgesics and within 30 min of administration of intravenous, intramuscular, or subcutaneous analgesics, or initiation of epidural/peripheral nerve infusion analgesics.

### 2.3. Chart Review Form and Patient/Caregiver Survey Instrument

The electronic medical record (EMR) review focused on demographics (age, sex, preferred language, admission diagnosis, length of stay, primary service, and location of care), pain assessment in the past 24 h (scales used, scores, number of assessments, re-assessments after intervention), pain interventions (medications with route and number of doses, non-pharmacologic/integrative modalities), and whether the PPPIM service had been consulted. Interventions for pain were classified as pharmacologic or non-pharmacologic/integrative. It was also noted if the admission pain assessment had been completed by the patient’s admitting nurse within 24 h of admission, as required by institutional policy (presence of pain on admission, type of pain, ways child communicates pain, pain regimens).

Pharmacologic interventions included pain medications divided into 3 categories: basic analgesia (e.g., acetaminophen or non-steroidal anti-inflammatory medications [NSAIDs]), opioids (excluding opioids received for sedation), and adjuvants (e.g., gabapentinoids, muscle relaxants). Non-pharmacologic/integrative interventions were categorized into five broad categories: local (e.g., heat/cold), distraction, emotional support, positioning, and relaxation techniques.

The patient/caregiver survey was adapted from previous surveys with permission [1,7]. The first survey item was a screening question to determine if the patient had experienced pain during the previous 24 h (yes/no). If the patient had not experienced pain, the interview was not undertaken. Pain intensity scores at the time of interview and their worst pain in the past 24 h were assessed using the NRS or FPS-R, depending on age. Patients/caregivers were also asked what caused the worst pain they experienced in the hospital, pain interventions received (i.e., analgesic medications, non-pharmacologic/integrative medicine therapies such as massage or aromatherapy), and their helpfulness (5-point Likert scale from very unhelpful to very helpful), satisfaction with pain management (5-point Likert scale from very dissatisfied to very satisfied), interventions that worked well, and what could have been done better. They were also asked about their pain prior to admission, including the intensity and cause of the worst pain they had experienced prior to admission.

### 2.4. Classification of Level of Pain

In order to standardize pain intensity categories across patients and to account for the variety of different pain scales used by hospital staff in different clinical situations, we classified pain as absent for scores of 0 or “none”; mild for scores of 1–3 on a numeric scale (i.e., NRS25, r-FLACC), FPS-R, NIPS, NPASS, or non-numeric verbal descriptors such as “none” or “mild” if a standardized pain scale was not used [1,3,6]. Pain was labeled “moderate” to “severe” for scores of ≥4 on a 0–10 scale, including the NRS-R, FLACC, FPS-R, NIPS [31], NPASS (pain component) [31,32], a “yes” answer on “assume pain is present”, or a verbal description indicating moderate or severe pain.

### 2.5. Patient/Caregiver Survey Administration

Children or their caregivers were invited to participate by a pediatric pain nurse if they were receiving care in one of eight inpatient units of the children’s hospital: two acute care, one hematology–oncology, one bone marrow transplant, and four ICUs. The survey was not announced to hospital staff to minimize the Hawthorne effect, whereby staff modify their practice knowing it is being observed. An attempt was made to interview all patients/caregivers present on the 8:00 am census and who had been admitted for at least 24 h. Patients/caregivers were interviewed only once, even if they remained on the census the next day. Patients were interviewed directly whenever possible if they were at least school-aged, agreed to participate in the survey, were developmentally able to understand and answer the questions, and if their clinical status allowed it. When those conditions were not fulfilled, the caregiver who was at the bedside was interviewed as a proxy for the child. Before the interview, the project was introduced to the patient and caregivers, and verbal consent and assent were obtained. Up to three attempts were made to locate the child and their caregivers over the course of the day. If a patient/caregiver was unavailable after three attempts, only the data from the chart review was used. The chart reviews and interviews were conducted by four pediatric pain nurses. A pilot run was conducted with 12 patients, followed by a debrief to reduce inter-rater variability and standardize data collection processes. Results of the survey and practice feedback were sent to all unit managers within two days.

### 2.6. Statistical Analysis

Descriptive data are presented as numbers, percentages, medians, and interquartile (IQR) ranges. A one-way analysis of variance (ANOVA) was run to examine the relationship between frequency of assessment and categorical (mild, moderate, severe) pain scores, and all other associations were examined with chi-square or Fisher’s exact tests. *p*-values < 0.05 were considered statistically significant. Patient/caregivers’ answers to open-ended questions were analyzed using qualitative content analysis [33]. Because chart data were collected by clinicians during usual clinical care, we were unable to control for missing data. No adjustments or imputations were made for missing qualitative data, resulting in different n’s for each analysis. Statistical analyses were performed using IBM SPSS statistical software (SPSS V.27 Inc., Armonk, NY, USA).

## 3. Results

### 3.1. Patient and Caregiver Characteristics

Among the 116 patients listed on the morning census, 107 had been admitted for at least 24 h. A total of 39 of 47 (83.0%) patients with documented pain were located for the survey, and 25 (53.2%) patients and caregivers completed it. Most interview respondents (*n* = 18, 69.2%) were parents (Table 1).

### 3.2. Pain Assessment

**Figure 1:**
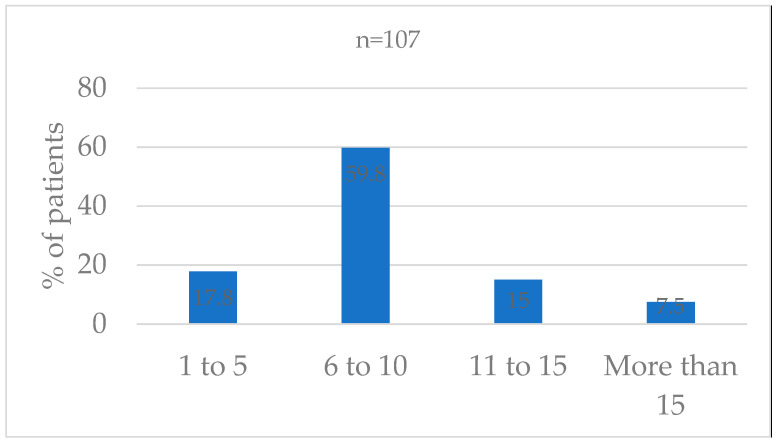
Number of assessments per patient documented in the past 24 h.

All 107 patients had at least one pain assessment in the previous 24 h. The median number of assessments per patient in the 24-h timeframe was 8 (range 1–22) (Figure 1).

A pain score of mild to severe was recorded in the chart ≥1 time in the past 24 h for 47/107 patients (43.9%), with the worst pain level in 24 h being mild in 12 (11.2% of all patients, 25.5% of patients with any pain) and moderate to severe in 35 (32.7% of all patients, 74.5% of patients with any pain). Children aged 6 months and older were statistically significantly more likely to have moderate to severe pain recorded in their chart (41.0%) compared to children younger than 6 months of age (21.7%) (x^2^ = 6.045, *p* = <0.05). The complete nursing pain assessment required on admission had been completed within 24 h of arrival for 49 (45.8%) patients. Of the 70 patients who had received at least one intervention for pain, the pain was re-assessed after each documented intervention in 20/70 (28.6%) patients. The more pain assessments a child had in the past 24 h, the more likely they were to have at least one moderate to severe pain score documented (yes/no) in the medical record (F = 8.229, *p* < 0.001).

### 3.3. Pain Interventions

Of the 107 total patients, 70 (65.4%) received ≥1 intervention targeting pain (medications and/or non-pharmacologic/integrative modalities), including most of those with any pain recorded in their chart (42/47, 89.4%), and 28/60 (46.7%) of those who only had pain scores of 0 recorded in their chart. A total of 24 of the 47 patients with at least mild pain (51.1%) received a combination of medications and non-pharmacologic/integrative therapies.

#### 3.3.1. Pharmacologic Pain Interventions

Pain medications were administered to 55 of 107 total patients (51.4%), including 39/47 patients with ≥1 mild pain score documented (83.0%). Many patients who received medications received ≥1 basic analgesic medication (*n* = 35/55, 63.6%), ≥1 opioid (*n* = 35, 63.3%), and 13 (23.6%) received ≥1 adjuvant (Table 2). Over half (*n* = 29/55, 52.7%) received multimodal pharmacologic treatment: a combination of 2 categories of pain medications (*n* = 25, 45.5%) or all 3 categories *(n* = 4, 7.2%). Over a third of patients receiving opioids did not receive basic analgesic medications (*n* = 15/35, 42.9%), and 9 (x/x, %) patients received pain medications despite having a maximum pain score of zero.

#### 3.3.2. Non-Pharmacologic/Integrative Pain Interventions

A total of 39 of 107 patients (36.4%) received ≥1 non-pharmacologic/integrative intervention, including nearly half *(n* = 23/47, 48.9%) of patients with a documented pain score of at least mild. Most patients who received non-pharmacologic/integrative therapies received at least one type (28/39, 71.8%): positioning/holding/environmental change (28 times; 51.9%), distraction/music (12 times; 22.2%), emotional support/presence (6 times; 11.1%), local modalities such as heat, cold, back rub, suction, oral care (6 times; 11.1%), and relaxation (2 times; 3.7%).

### 3.4. Pediatric Pain, Palliative and Integrative Medicine Consultation

Approximately one-third of patients with any documented pain received a PPPIM consultation (*n* = 17/47, 36.2%), and 11/35 (31.4%) with moderate to severe pain received a PPPIM consultation. Table 3 shows the characteristics of the 24/35 (68.6%) patients with moderate to severe pain who did not receive a PPPIM consultation. Of those patients, 15 (62.5%) were 5 years of age or younger. Nearly two-thirds of the 24 patients who did not receive a consultation were cared for by intensive care unit or hematology–oncology/bone marrow transplant teams (*n* = 8/24 (33.3%) and *n* = 7 (29.2%), respectively). Non-pharmacologic/integrative modalities were documented in 11 (45.8%) of these patients. One-third (*n* = 8, 33.3%) reported that their worst pain was related to a painful procedure or surgery.

### 3.5. Patient Pain Experiences

A total of 39 patients/caregivers were approached and screened for an interview, 25 (64.1%) of whom reported pain in the past 24 h and were interviewed. The worst pain level reported by these patients/caregivers was mild (*n* = 10, 40.0%), moderate (*n* = 10, 40%), and severe (*n* = 5, 20%). Ten (40%) reported multiple sources of pain in the past 24 h. The most common causes of worst pain cited were acute illness (*n* = 13, 52%), procedures (*n* = 10, 40%) such as needle pokes, dressing change, or central line placement, and surgery (*n* = 2, 8%). There was no significant association between the categorical (mild, moderate, severe) level of worst pain documented in the chart and the level of worst pain reported by patients/caregivers.

When asked which interventions worked well for their pain, 10/25 patients did not answer; of the 15 patients who responded, 6 (40.0%) cited a combination of pharmacologic and non-pharmacologic/integrative modalities, 5 (33.3%) cited pharmacologic only, 3 (20%) cited non-pharmacologic/integrative modalities only, and 1 (6.7%) cited communication. When asked what could have been done better regarding their pain management, nearly half (*n* = 9/21, 42.9%) of patients/caregivers who responded answered “nothing”, 5 (23.8%) said they would have liked more integrative modalities, 4 (19%) mentioned improving communication around pain, 2 (9.5%) said optimizing pain management around procedures, and 1 (4.8%) said improving medication side effect management. Most patients/caregivers were satisfied or very satisfied with pain control (*n* = 20, 87.0%); only 3 (13.0%) were neutral or dissatisfied.

## 4. Discussion

This mixed-method, cross-sectional survey at a tertiary children’s hospital showed that all 107 children admitted for at least 24 h had at least one pain evaluation documented in their chart. Fifty-six percent of patients had no recorded pain; however, most of the patients who had documented pain had scores indicating moderate to severe pain. About 90% of patients who had experienced mild, moderate, or severe pain in the previous 24 h received a pain intervention; however, multimodal analgesia and non-pharmacologic/integrative modalities, and PPPIM consultations were under-utilized. Painful procedures were cited by parents/caregivers as the source of worst pain by over a third of patients.

These findings establish a benchmark of pain assessment, prevalence, intensity, documentation, and treatment in children admitted to our institution, which is helpful in comparing our pain management practices with other children’s hospitals and serves as a pre-COVID-19 pandemic baseline for upcoming QI efforts at our campuses. Despite some encouraging findings compared to prior surveys, these data also highlight the areas for improvement that remain at a tertiary center with a pediatric pain management service, despite literature on practice gaps published by other authors over the past two decades.

The rate of pain documentation in this study is higher than previously reported in the literature. [1,2,3,4,6,7,8,9,10]. However, despite the documentation rate, we noticed two areas where documentation was suboptimal. First, the nursing pain assessments on admission, as required by policy, were completed within 24 h of arrival in only half of patients. Second, a re-assessment of pain was documented after each intervention in less than a third of patients. Furthermore, there were indirect signs of incomplete documentation of pain scores, such as medications ordered for as-needed use for pain (including opioids, suggesting more severe pain) even when pain scores were zero. Interestingly, we found that children with more frequent assessments were significantly more likely to have at least one moderate to severe pain score (*p* < 0.001). This could suggest that the more pain is evaluated, the more likely that a child has moderate to severe pain or that the more pain a patient is experiencing, the more likely they are to have frequent pain assessments. There are no evidence-based standards regarding the frequency of pain assessment, but our institution’s guidelines require a focused pain assessment with vital signs every 4 h, before and after any pain treatment, and as needed based on patients’ conditions. Other institutions’ online guidelines recommend evaluating and documenting pain at least once a shift, and more often for patients who had recent surgery, have pain, or are receiving an opioid infusion or epidural medications [34].

We found that nearly half (44%) of patients and parents/caregivers reported a pain score > 0 in the previous 24 h, and about 3/4 (74.5%) of those patients had moderate to severe pain. Compared to prior studies, the prevalence of documented overall pain and moderate to severe pain were higher (74.5% vs. 24–82%) [1,2,3,5,6,7,8,9,11]. In other words, pain was documented for few patients, but when it was, it was more often moderate to severe in intensity. This could be due to mild pain being less likely to be recorded in the EMR. It is possible we did not include all children in the survey who actually had pain during the past 24 h, thus underestimating pain, which is well described in the literature [1,3,6,7,35,36,37,38,39,40]. This may also have contributed to the lack of association between the severity of self-reported pain scores and pain scores documented in the EMR.

Our data suggest that painful procedures were likely an under-documented contributor to severe pain. In our sample, 40% of respondents attributed their worst pain to painful procedures, which is consistent with ranges of 34–40% reported in prior studies [7,8,9,11]. Furthermore, one-third of patients with moderate to severe pain who did not receive a PPPIM consultation reported that their worst pain was related to a painful procedure or surgery, which was not evident upon chart review. Altogether, these findings suggest that procedural pain might be overlooked as a cause of significant pain and likely is under-documented and under-treated, as described in prior studies [1,3,4,6,7,8,9,10,11]. These findings highlight the need for standardized policies with consistent implementation for painful procedures. We are currently preparing to implement an institution-wide QI project aimed at preventing pain for the most common painful procedure: needle procedures. We will offer a bundle of interventions (the “Comfort Promise”: topical anesthetic, sucrose/breastfeeding, positioning, distraction) to children every time they undergo a needle procedure, similar to another US children’s hospital [23].

Survey findings indicated 89% of children with documented pain received an intervention for pain (pharmacologic and/or integrative), and 83% of children with documented pain received pain medications, indicating that pain was untreated, similar to findings from prior studies [1,3,7,8,9,10]. While these results are encouraging, we must strive to prevent and treat pain 100% of the time. To address pain, a multimodal approach, including non-pharmacologic/integrative therapies, is often the most effective [41]. Our work is among the few recent surveys [4,6,7,8,9,10] examining the use of non-pharmacologic/integrative modalities in greater detail. Despite 60% of interviewed patients citing multimodal and/or non-pharmacologic/integrative modalities as the most helpful in treating their pain, only half of patients with pain (51.0%) received a combination of pharmacologic and non-pharmacologic/integrative therapies. These findings suggest that we should strive to increase the use of multimodal strategies combining pharmacologic and non-pharmacologic/integrative strategies. Since this survey was conducted, we have implemented several QI measures, including launching a new order set to encourage the use of multimodal analgesia and opioid-sparing analgesic medications, improving access to non-pharmacologic/integrative modalities by expanding the integrative arm of our PPPIM service, and facilitating clinicians’ direct access to aromatherapy.

Finally, three-fourths of patients with moderate to severe pain did not receive a PPPIM consult, suggesting underutilization. Patient characteristics that may have contributed to underutilization included age and referring unit. Specifically, patients with moderate to severe pain without a consult were either very young (40% were below 1 year of age) or older (29% were teenagers and young adults), which might reflect under-recognition of moderate to severe pain and its impact on patients in those age groups. They were also mostly cared for in intensive care units or oncologic/bone marrow transplant units, which is likely a reflection of their disease severity and complexity or their subspecialists’ perception, experience, and/or level of comfort managing complex pain. These findings draw our attention to profiles of patients who are more likely to experience moderate to severe pain and may benefit from a specialized consultation. They also help us target our efforts to increase awareness of our teams’ spectrum of pain management services and the benefits of consultation.

### Limitations

This survey has several limitations. First, our survey may paint an incomplete picture of our patients’ experiences with pain during their admission, with a risk of underestimating pain, due to several factors. Only patients who had at least mild pain recorded in their chart were interviewed, and reliance on likely incomplete (and sometimes evidently inaccurate) EMR data implies that patients with pain are likely to have been missed. As a result, only one-fourth of admitted patients were interviewed. Additionally, over 80% of interview respondents were caregivers rather than patients themselves, as many patients could not be interviewed directly due to being too young or too sick. It has also been shown that both clinicians and caregivers widely underestimate pediatric patients’ pain, suggesting our pain prevalence survey findings may be lower than actual pain prevalence [1,3,7,35,36,38,39,40]. Furthermore, painful procedures are often undertaken when caregivers are not present (e.g., blood draws early in the morning), potentially resulting in underreporting in this survey. However, because of our retrospective survey design, we are unable to identify the cause of missing data. This may have contributed to the small number of statistically significant associations. Additionally, patients could have under-reported pain or dissatisfaction to the surveyors, who were nurses with the pain team, for fear of compromising their care. Second, there was wide variation in the types of scales used by the different units of our hospital, requiring us to convert all scales to three categories of pain severity (no pain, mild pain, moderate to severe pain). This may have resulted in loss of granularity of the data, especially the inability to identify patients with severe pain. Additionally, pain scores alone might not be optimal for identifying clinically significant pain, as levels of acceptable pain vary from person to person [4,42]. Third, our survey did not include patients admitted to the well-baby nursery, emergency department, post-anesthesia care unit, or visiting outpatient clinics and labs, where patients routinely undergo painful procedures such as circumcisions, suturing, and phlebotomy. Therefore, we may have missed patients with significant pain, resulting in an underestimation of hospital-wide pain prevalence and severity.

## 5. Conclusions

Our survey findings indicate that all patients admitted to our children’s hospital for at least 24 h had at least one pain score recorded in their medical record and that most patients with pain received a pain intervention. Multiple areas for improvement were found, including consistency of pain documentation, use of routine assessment, greater use of multimodal treatment strategies, including nonpharmacologic approaches, improving adherence to established hospital pain protocols, and ensuring high visibility of pediatric specialty pain services for children with clinically significant pain.

## Figures and Tables

**Table 1:** Patient characteristics.

	*n* (%)
Admitted > 24 h	107
Length of stay in days: Median (min, max)	15 (1, 176)
Age	
<6 months	46 (43.0)
6 months–2 years	11 (10.3)
3–6 years	12 (11.2)
7–12 years	14 (13.1)
13+ years	24 (22.4)
Sex	
Female	57 (53.3)
Male	50 (46.7)
Language	
English	99 (92.5)
Spanish	5 (4.7)
Other *	3 (2.8)
Location of care	
Neonatal ICU	29 (27.1)
Pediatric ICU (including cardiac)	31 (29.0)
Acute care units (medico-surgical, transitional care unit)	30 (28.0)
Hematology–oncology	17 (15.9)
Admission diagnosis	
Hematologic–oncologic	16 (15.0)
Cardiovascular	19 (17.8)
Newborn-related, non-cardiovascular	28 (26.2)
Neurological	11 (10.3)
Respiratory	7 (6.5)
Other systems (endocrine, renal, GI)	25 (23.4)
Unknown	1 (0.9)
Interviews	
Attempted	39 (36.4)
Patient/caregiver endorsed no pain in past 24 h	13 (12.1)
Patient had pain and survey was answered	25 (23.4)
Respondent	
Mother	14 (53.8)
Father	2 (7.7)
Both parents	2 (7.7)
Patient	5 (19.2)
Other **	2 (7.7)

* Other languages: Tagalog, Tigrinya, unknown. ** Other respondents: aunt, unknown.

**Table 2:** Pharmacologic Pain Management Interventions in The Past 24 h in The 55 Patients Who Received Analgesics.

	*n* (%)
**Basic analgesia**	35 (63.6)
Acetaminophen	33 (60.0)
Ibuprofen	4 (7.3)
Ketorolac	3 (5.5)
Naproxen	1 (1.8)
**Opioids**	35 (63.6)
Morphine	14 (25.5)
Fentanyl	1 (1.8)
Hydromorphone	11 (20.0)
Methadone	3 (5.5)
Oxycodone	10 (18.2)
**Adjuvant analgesia**	13 (23.6)
Gabapentin	6 (10.9)
Topical lidocaine	3 (5.5)
Cyclobenzaprine	1 (1.8)
“Magic Mouthwash” (lidocaine, diphenhydramine, antacid)	1 (1.8)
Ketamine	1 (1.8)
Sucrose	1 (1.8)

**Table 3:** Patients with moderate to severe pain who did not receive a PPPIM consultation.

Patient No.	Age (Years or Months)	Sex	Admission Diagnosis	LOS (Days)	Primary Team	Pain Severity, Patient Report	No. Pain Assessments	Analgesics	Integrative Modalities	Painful Procedure
2	8 m	M	Sepsis due to MSSA	18	PICU	Mild	15	Y	Y	Yes (needle pain)
3	10 y	F	Tachycardia	5	PICU	N/A	8	Y	N	Unknown
5	2 m	M	Congenital ventriculomegaly of brain	1	PICU	N/A	14	Y	Y	Yes (surgery)
6	6 m	F	Tachypnea	4	PICU	Mild	6	Y	Y	Unknown
7	3 m	M	Polycystic renal disease with renal failure	83	PICU	None	11	Y	N	Yes (skin biopsy)
16	3 y	M	Chronic granulomatous disease with feeding difficulties	2	PHM	None	7	Y	N	Unknown
34	15 y	F	Focal epilepsy	2	PHM	N/A	10	Y	N	Unknown
35	14 y	F	Partial epilepsy	2	PHM	N/A	9	Y	N	Unknown
65	18 y	M	Acute lymphocytic leukemia pre-bone marrow transplant	8	BMT	Moderate	8	Y	N	Unknown
70	19 y	F	Desmoplastic small cell tumor post-bone marrow transplant	15	BMT	N/A	13	Y	N	Unknown
71	4 y	F	Juvenile myelomonocytic leukemia	50	BMT	N/A	14	Y	Y	Unknown
72	10 y	M	Cystitis post-bone marrow transplant	4	BMT	Moderate	7	Y	Y	Unknown
73	2 m	F	Severe combined immunodeficiency pre-bone marrow transplant	47	PHM	Severe	7	Y	N	Unknown
74	20 y	M	T cell leukemia	12	HO	Severe	8	Y	N	Yes (lumbar puncture)
75	3 yo	M	Medulloblastoma	15	HO	Moderate	12	Y	Y	Unknown
81	5 y	F	Graft-versus-host disease complicating bone marrow transplant	34	BMT	N/A	9	Y	N	Unknown
86	12 y	F	Pulmonary stenosis	2	CTCU	Mild	8	Y	Y	Yes (chest tube insertion)
88	5 m	F	Ventricular septal defect	6	CTCU	N/A	11	Y	N	Unknown
95	9 m	M	Alagille syndrome post-liver transplant	13	TCU	None	8	Y	Y	Unknown
96	18 y	M	End-stage renal disease post-kidney transplant	6	TCU	Moderate	8	Y	Y	Yes (Foley catheter removal)
99	3 m	M	Hydrocephalus complicated by seizures	8	TCU	N/A	8	Y	Y	Unknown
127	2 m	M	Tracheo-esophageal fistula	63	CICU	Mild	16	Y	Y	Yes (surgery)
128	3 y	F	Atrioventricular canal post-repair	10	CICU	Moderate	9	Y	Y	Yes (surgery)
129	1 m	M	Truncus arteriosus	35	CICU	N/A	15	N	Y	Unknown

## Data Availability

The raw data supporting the conclusions of this article will be made available by the authors on request.
